# Complete mitochondrial genome of the red-eared slider (*Trachemys scripta elegans*, Testudines: Emydidae) in Korea

**DOI:** 10.1080/23802359.2021.1887773

**Published:** 2021-03-17

**Authors:** Jaehong Park, Jae-I Moon, Yun-Ju Song, Seung-Min Park, Seungju Cheon, Ha-Cheol Sung, Dong-Hyun Lee

**Affiliations:** a School of Biological Sciences and Biotechnology Graduate School, Chonnam National University, Gwangju, Korea; b Research Center of Ecomimetics, Chonnam National University, Gwangju, Korea; c Department of Biological Sciences, College of Natural Sciences, Chonnam National University, Gwangju, Korea

**Keywords:** *Trachemys scripta elegans*, Emydidae, mitochondrial genome

## Abstract

The complete mitochondrial (mt) genome of *Trachemys scripta elegans* in Korea was sequenced and characterized. The mt genome is constituted of 37 genes (13 protein-coding genes, 22 transfer RNA genes and 2 ribosomal RNA genes) and a noncoding control region. Phylogenetic analysis based on the complete mt genome showed that *T. s. elegans* Korea has closer relationship with *T. scripta* Canada than *T. s. elegans* China. This is the first complete mt genome from *T. s. elegans* in Korea, which provides information for biogeographical studies and management plan for invasive species.

The Red-eared slider (*Trachemys scripta elegans*) is native to the United States (Parham et al. 2020). With rapid increase in the turtle’s pet purchase through international trades and intentional release, the number of exotic turtles found in the wild has increased. Thus, this turtle has been designated as the invasive species in International Union for Conservation of Nature (ICUN; http://www.iucn.org) (Parham et al. 2013). The species would compete with native species, preoccupy prey, transfer diseases, and thus reduce the number of native species (Hayes et al. 2018, Koo et al. 2020). In addition, the turtles can cause genetic contamination of native species through hybridization with native species (Parham et al. 2013). The import of red-eared sliders for pet into Korea began in the late 1980s at first (Koo et al. 2020). While non-native red-eared sliders have inhabited in Korea for about 40 years, genetic variation could occur in their genome. This variation is expected to be distinct from that of red-eared sliders in original habitats or other nations. Nonetheless, there is no genetic information of red-eared sliders (*T. s. elegans*) in Korea, and just only one study about the complete mitochondrial (mt) genome of the red-eared slider (*T. s. elegans*) was reported in China (Yu et al. 2016). Here, We sequenced the complete mt genome of *T. s. elegans,* inhabiting Korea. This analysis can contribute to phylogenetic and evolutionary studies.

The *T. s. elegans* specimen was collected from Gwangju (35°10′28.1″ N; 126°54′35.9″ E), Korea, and the total genomic DNA was extracted from the tail using the DNeasy Blood & Tissue kit (Qiagen, Valencia, CA) according to the manufacturer’s protocol. The extracted DNA sample was deposited at the Museum of Wildlife, located in Research Center of Ecomimetics, Chonnam National University, Korea (Specimen accession number: 2020-RCE-TSE001; shcol2002@chonnam.ac.kr). The mt genome was determined by primer walking method and sequenced using Applied Biosystems 3730XL DNA Analyzer (Applied Biosystems, Foster City, CA), which was performed by Bionics (Seoul, Korea). The reads were aligned and the complete sequence was annotated by comparing GenBank data (FJ392294, KM216748, and KM216749).

The complete mt genome of *T. s. elegans* is 16,808 bp in length deposited in GenBank (Accession number: MW019443), and contains 13 protein-coding genes, 22 transfer RNA (tRNA) genes, 2 ribosomal RNA (rRNA) genes, and a putative long non-coding control region. 12 protein-coding genes, 14 tRNA genes, and 2 rRNA genes are encoded in heavy strand, whereas 1 protein-coding gene (NADH dehydrogenase subunit 6) and 8 tRNA genes in light strand. The nucleotide composition of the *T. s. elegans* Korea mt genome (A = 34.3%, T = 27.0%, C = 25.9%, and G = 12.9%) is identical with that of *T. scripta* Canada (A = 34.3%, T = 27.0%, C = 25.9%, and G = 12.9%), and almost identical to that of *T. s. scripta* China (A = 34.2%, T = 27.0%, C = 25.9%, and G = 12.9%) and *T. s. elegans* China (A = 34.2%, T = 27.0%, C = 25.9%, and G = 12.9%). The sequence identity between *T. s. elegans* Korea and *T. scripta* Canada was 99.9%, but the sequence comparisons between *T. s. elegans* Korea and China indicated a 99.6% sequence identity.

To investigate the phylogenetic position of *T. s. elegans* Korea, the complete mt genome sequences of 12 species in *Testudines* were extracted from GenBank and the phylogenetic tree was constructed using MEGA X software (Figure 1). Tree building was performed using the maximum likelihood method and Tamura-Nei model (Tamura and Nei 1993). In the phylogenetic tree, every *T. scripta* species is clearly clustered in monophyletic manner, and separated completely with other species (*Mauremys reevesii, Mauremys sinensis, Chrysemys picta, Macrochelys temminckii,* and *Chelonia mydas*). And, *T. s. elegans* Korea shares closer relationship with *T. scripta* Canada than with *T. s. elegans* China. *T. s. elegans*, as the invasive species, spreads worldwide and has a negative effect on ecosystem. Therefore, it should be controlled for preservation of native species and biodiversity. These data provide important molecular data for further biogeographical studies. And they can be utilized meaningfully to control ecological disturbance by *T. s. elegans* which is an invasive species in many countries including Korea.

**Figure 1.**
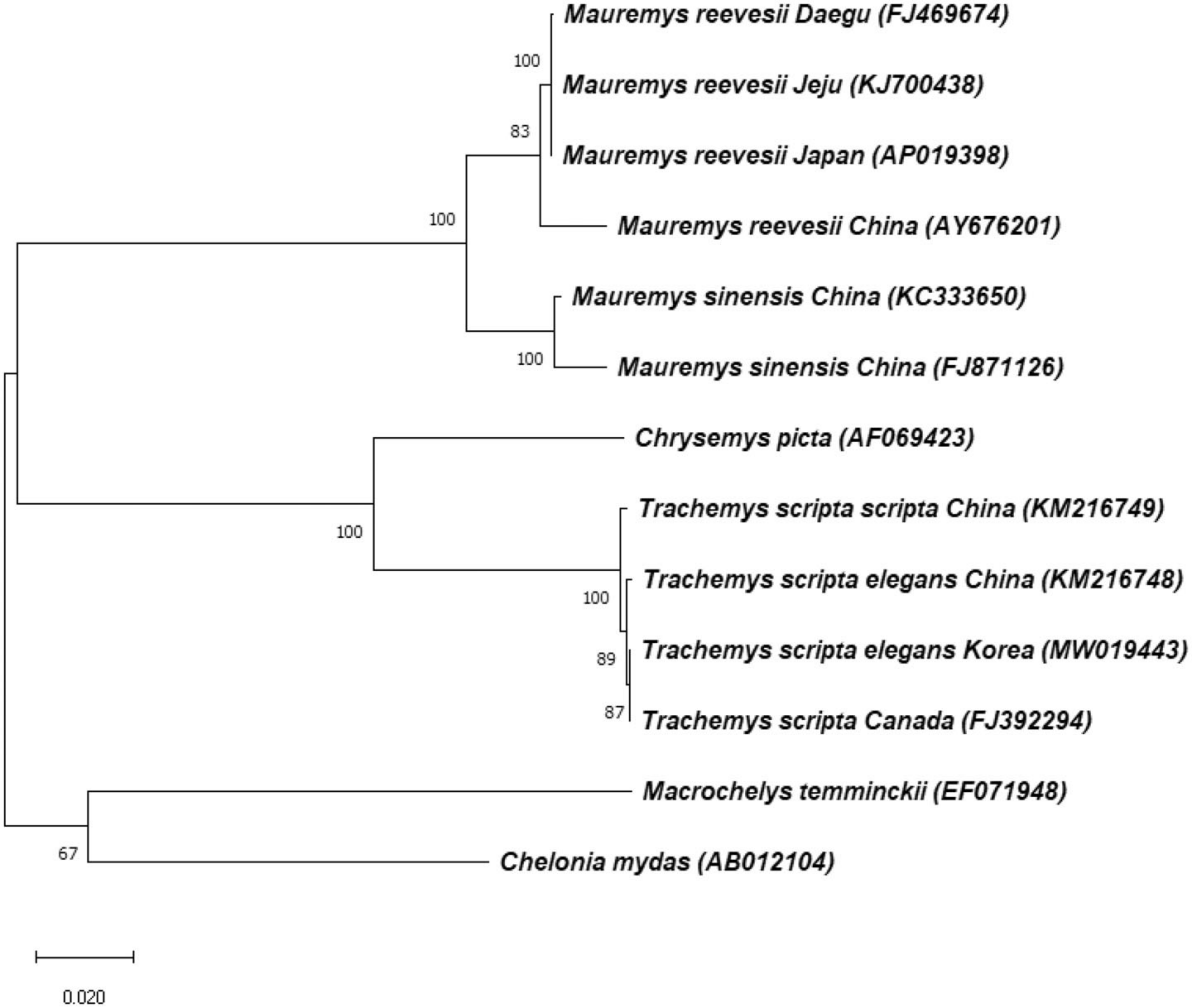
Phylogenetic tree of *Trachemys scripta elegans* Korea and other related species based on complete mt genome sequences. The complete mt genomes were extracted from GenBank and the phylogenetic tree was constructed by a maximum-likelihood method with 1,000 bootstrap replicates. GenBank accession numbers of each mt genome sequence are given in the bracket after the species name.

## Data Availability

GenBank accession number from the complete mitochondrial genome of *Trachemys scripta elegans* (MW019443) has been registered with the NCBI database (https://www.ncbi.nlm.nih.gov/).
